# Positive and negative aspects of bacteriophages and their immense role in the food chain

**DOI:** 10.1038/s41538-023-00245-8

**Published:** 2024-01-03

**Authors:** Soniya Ashok Ranveer, Vaishali Dasriya, Md Faruque Ahmad, Harmeet Singh Dhillon, Mrinal Samtiya, Eman Shama, Taruna Anand, Tejpal Dhewa, Vishu Chaudhary, Priya Chaudhary, Pradip Behare, Chand Ram, Dharun Vijay Puniya, Gulab D. Khedkar, António Raposo, Heesup Han, Anil Kumar Puniya

**Affiliations:** 1 Dairy Microbiology Division, ICAR-National Dairy Research Institute, Karnal, 132001 India; 2 Department of Clinical Nutrition, College of Applied Medical Science, Jazan University, Jazan, 45142 Saudi Arabia; 3 Department of Nutrition Biology, School of Interdisciplinary and Applied Sciences, Central University of Haryana, Mahendergarh, 123031 India; 4 ICAR-National Research Centre on Equines, Sirsa Road, Hisar, 125001 India; 5 University Institute of Biotechnology, Chandigarh University, Sahibzada Ajit Singh Nagar, 140413 India; 6 Microbiology Department, VCSG Government Institute of Medical Science and Research, Ganganali Srikot, Srinagar Pauri Garhwal, 246174 India; 7 Centre of One Health, College of Veterinary Science, Guru Angad Dev Veterinary and Animal Sciences University, Ludhiana, India; 8 Paul Hebert Centre for DNA Barcoding and Biodiversity Studies, Dr. Babasaheb Ambedkar Marathwada University, Aurangabad, India; 9 CBIOS (Research Center for Biosciences and Health Technologies), Universidade Lusófona de Humanidades e Tecnologias, Campo Grande, 376 1749-024 Lisboa, Portugal; 10 College of Hospitality and Tourism Management, Sejong University, 98 Gunja-Dong, Gwanjin-gu, Seoul, 143-747 Republic of Korea

**Keywords:** Biofilms, Antimicrobials

## Abstract

Bacteriophages infect and replicate inside a bacterial host as well as serve as natural bio-control agents. Phages were once viewed as nuisances that caused fermentation failures with cheese-making and other industrial processes, which lead to economic losses, but phages are now increasingly being observed as being promising antimicrobials that can fight against spoilage and pathogenic bacteria. Pathogen-free meals that fulfil industry requirements without synthetic additives are always in demand in the food sector. This study introduces the readers to the history, sources, and biology of bacteriophages, which include their host ranges, absorption mechanisms, lytic profiles, lysogenic profiles, and the influence of external factors on the growth of phages. Phages and their derivatives have emerged as antimicrobial agents, biodetectors, and biofilm controllers, which have been comprehensively discussed in addition to their potential applications in the food and gastrointestinal tract, and they are a feasible and safe option for preventing, treating, and/or eradicating contaminants in various foods and food processing environments. Furthermore, phages and phage-derived lytic proteins can be considered potential antimicrobials in the traditional farm-to-fork context, which include phage-based mixtures and commercially available phage products. This paper concludes with some potential safety concerns that need to be addressed to enable bacteriophage use efficiently.

## Introduction

Phages have been discovered in every environment where bacteria can grow, but there hasn’t been much research into their ecological importance in the biosphere^1^. Phages eliminate approximately 40% of bacterial biomass daily^2^. Studies on the effects of phages on cohabiting microorganisms remain rare and undervalued, even in the most complex ecosystems. The most studied environments, which include food processing, human guts, and plant crops, have a lot to learn in terms of their environmental phages and their impact in various contexts. Also, a lot of work is still needed to employ phages in medical and biotechnological applications. The prevention and treatment of infectious diseases in humans, animals, and plants remain the primary objectives of bacterial virus research^3^. The rise of multidrug-resistant human infections and the emerging concerns of antibiotic resistance among pathogenic bacteria have renewed interest in phage therapy. Viruses could be used alone or in combination with other viruses in order to reduce infections in situ. These studies focus on pathogenic and spoilage-causing bacteria^4^. A phage is a promising new weapon in the fight against antibiotic-resistant, pathogenic, and biofilm-forming bacteria. There are several growth limitations to consider when using a bio-control approach regarding pH, temperature, and ion concentration. The virus heat stability is critical when using phages to control harmful microorganisms in food products. The temperature component of phage fitness should remain critical when considering the use of viruses in order to manage bacterial infections in agriculture and/ or the environment because these phage-based products may have inconsistent activity on the same disease, which is due to differences in climatic conditions, such as temperatures. Phages can act as allies and enemies in human activities, and bacteria may evolve phage resistance via different defense mechanisms^5^. The release of substances that prevent phage attachment to the bacterial pathogen, hiding, modifying, or removing the receptor, blocking phage DNA injection into a cell, altering or removing the receptor, and blocking phage replication and release are all examples of inhibiting phage replication and release. Antibiotic resistance genes (ARGs) may spread as a result of the horizontal gene transfer activities, which is due to their involvement in these processes. The bacteriophage poses a challenge to the fermentation economy since phage contamination can cause it to lyse an entire batch^6^. Bacteriophages can be employed to make food safer, and they can be used as an adversary in specific stages of food production. Disinfecting equipment and surfaces used in the food industry with bacteriophage biocontrol is an intriguing possibility^7^. The novel applications of phages in the food and allied industries and the currently available mixtures that describe the applications of these phages represent a significant gap in the literature about this topic, which is explained in this study in a detailed perspective. This will attract the readers and researchers working on bacteriophages and the related fields, because this review presents a broad overview of the topics, which are not previously comprehensively explained elsewhere. A summary of the businesses and commodities that use bacteriophages for food-safety purposes is also included.

## History, sources, and biology of phages

Bacteriophages were first studied and independently characterized by Felix d’Herelle in 1917, and Hankin and co- workers discovered that bacteriophages have a bactericidal effect on bacteria^1,8^. d’Herelle wrote several papers in the 1920s on bacteriophage biology, and he was credited with helping in regards to establish the International Bacteriophage Institute in Tbilisi, Georgia in 1923^9^. Phages are viruses that can only infect bacteria and are nearly 50 times smaller than bacteria (20–200 nm). Also, they can be found in soil, water, and a range of food products. Phages are categorized into various classes based on their size and morphology. Most of them have tails, but filamentous and pleomorphic phages can also be found in some phage families. Bacteriophage virion has the two most important components. They include nucleic acid, which is double-stranded or single-stranded DNA or RNA, and the protein envelope. Lipids are the constituents of the envelope or a specialized lipid wall in some cases^10^.

Phages have a high degree of selectivity when infecting the host bacteria. Phages are divided into two types: lytic, which is virulent, and lysogenic, which is temperate phages. The host cell is disrupted by lysis during the lytic cycle, which results in the cell’s death. Lytic phages are absorbed outside the host at a specific receptor site, later followed by a permanent attachment. The ability of a bacteriophage to effectively recognize and bind to the receptor molecules on the cell’s surface determines its host spectrum. Phages enter the bacteria’s cell walls with the help of their tails, which causes phage DNA to be inserted into the host cytoplasm. Unique enzymes encode the phage genome within the bacterial cell in order to produce a new phage particle and diversify the host cell’s DNA and protein production. It comprises the structural and enzymatic phage proteins that are necessary for cell lysis and the release of progeny phage. New phage particles are phage-encoded structural elements, and newly replicated viral genomes are bundled into phage heads^11^. Temperate phages are phages that can choose between the lytic and lysogenic growth pathways. These viruses can facilitate the transfer of genomic material from one bacterial cell to another, so these viruses are normally avoided as direct-use treatments. Temperate phages use lysogenic conversion, which is the transmission of genes that improve the pathogenicity of the host cell. As a result, these viral viruses are unable to kill the host that they infect. Furthermore, *super-infection immunity* occurs when a bacterial cell protects a prophage within its genome and develops a resistance to infection by comparable or nearly related phages^12,13^.

Pathogens are entered into the food system by non-food resources, such as wastewater from municipalities, faeces, soil, farms, and effluent processing facilities in food manufacturing plants. Pathogenic phages in food have been shown to be a lot more important than coliform phages and psychrotrophic spoilage phages^14^.

Isolating phages against various foodborne spoilage bacteria and indicator species is a lot more common than isolating phages against specific infection-causing bacteria^15^. Pattern-based studies that analyze the phage types of bacterial pathogens should be periodically conducted to identify and detect the changes in the phage biotypes^16^.

### Host range of phages

The host range of a phage is a challenging attribute to assess when determining its utility regarding phage therapy. The host range is defined by Hyman,^13^ as bacteria that are capable of supporting phage infections that stimulate the spread of new phage virions. The wider the host range within a target pathogen, the more probable a particular phage will be exploited by that same target pathogen in order to cause a specific disease. A phage should not infect other species because it may kill non-pathogenic commensal bacteria and dilute the phage’s optimum dosage toward the targeted bacteria. However, the condition will be much more composite if the non-target bacteria’s disease is productive. Other limits on the host’s range include bacterial anti-phage defenses, such as toxin-antitoxin systems, CRISPR and restriction enzymes are sometimes assumed to be a function of the precise receptor that is present in the targeted bacteria^13,17^. Phage systems are contradictory, and they also have a host range, a dynamic attribute that can alter over time^18^. There are various ways to determine the host range. Some of them are more precise and realistic than others. This approach is used to assess the host range using individual host species in groups for each phage that is being tested^19^. Diverse host range bacteriophages have been isolated and characterized, which can potentially prevent diarrhea in cattle^20^.

### Absorption mechanism

The main phase in bacteriophage (phage) infection on a competent host cell is the attachment of phage virions. Mass-action kinetics, which assume an influential effect of the host density and adsorption speed on the adsorption process, are commonly used in order to describe this adsorption mechanism. As a result, a high host density environment may be considered comparable to a phage capable of a high adsorption rate and vice versa. Phage strains with a greater adsorption rate will have a smaller optimal lysis period and vice versa.

### Lytic cycle

**Fig. 1:**
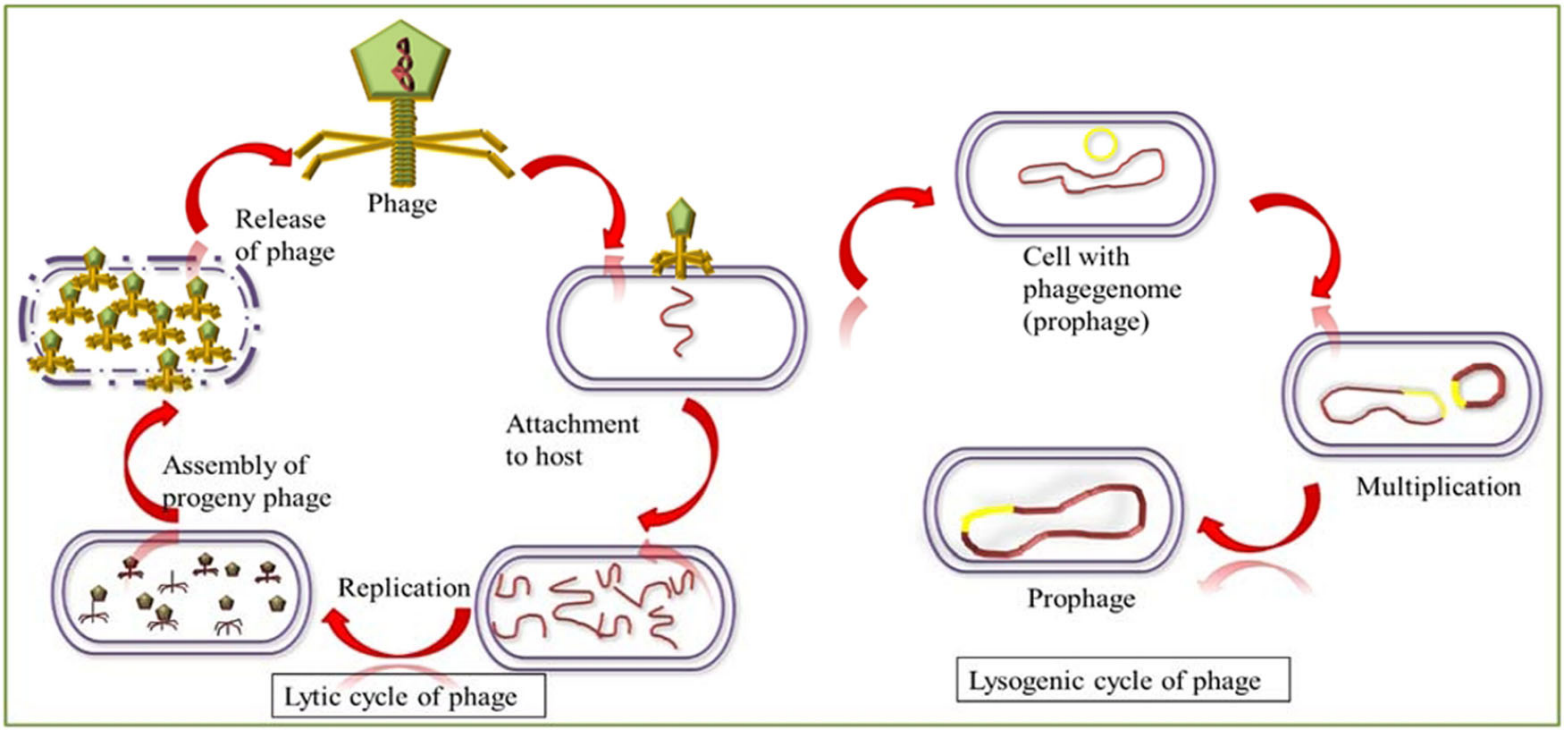
Life cycle of phages.

Only lytic viruses are used in phage therapy because these attack a cell from the outside and do not integrate their genomic material into the host cell’s DNA, which is due to their shorter replication cycle. Lytic viruses change their hosts’ genomes so they can multiply and spread new viruses by bursting or lysing the cell membrane after a certain amount of time. These new viruses infect and propagate quickly in order to infect the nearby cells^21^. (Fig. 1).

### Lysis profile

The isolated phages are examined for their ability to infect host bacterial cells in order to assess their infectivity range or lysis efficacy. The ability of the phage to form a clear plaque, a muddy plaque, or no lysis against a given host cell is used to make this determination. Bacterial cultures are cultivated on a special agar medium, and phage lysates are used to pattern the lawns. Plaques are detected by incubating the plates at certain temperatures and periods. Liquefaction profiles, plaque clarity, and size are used in order to determine the most efficient phages with the zones of lysis indicating bacteriophage lysis patterns^22^.

### Lysogenic cycle

Viruses that infect the cells and incorporate their genetic material into the host genome cause eternal association as a *prophage* with the cell and all its progeny. Its genome is retrieved in intervals from the host and starts replication, and as a final point, it breaks open the cell and sets free the new viruses (lysis). The lysogenic cycle extends the infection of the virus over several replications of the infected host cell. The parts of the bacterial DNA are sometimes carried along through the viral genome during the cutting-out process. The spreading of this type of genome and its exchanging property may permit the *transduction* in the infected bacterial cell, which plays the main role in the bacterial resistance properties. Hence, lysogenic phages are incompatible candidates for phage therapy due to their elongated infection cycle^23^.

## Influence of external factors on phages

Researchers have evaluated the impact of physical and chemical parameters, which include temperature, acidity, and ions, on phage persistence. Phage sensitivity is an intricate issue that necessitates a detailed study of the involved external environmental elements. Understanding the difficulties could be beneficial to people interested in phage pharmacology and agriculture as well as to people who deal with them^24^. Damage to the phage’s internal structures, which include the envelope, head, and tail, the loss of lipids, and/or DNA damage can render the virus inactive. Large capsid phages with a diameter of 100 nm will survive better than tiny capsid phages with a diameter of 60 nm. Still, there is no substantial difference in the sensitivity between contractile, non-contractile, and short tails in adverse conditions^10^. A phage’s characteristics and tolerance to external pressures may not be determined by close structural similarities or family members, and they may depend on other variables^25^.

### Temperature

Temperature plays a vital role in a phage’s survival. The lysogenic phase cycle is dominated by attachment, penetration, multiplication, and the duration of the latent phase temperature. Only a few phages genetic materials can infiltrate the host cell when the temperature is below the optimum temperature, and only a few phages participate in phage multiplication^26^. Tey et al.^27^ discovered that phages that are kept at a high temperature could extend the latent stage. Furthermore, the speed, viability, and storage of phages are all influenced by temperature. Phages may thrive in hot springs, which are uncommon habitats with temperatures that range from 40 to 90 °C. Phages were isolated from hot springs in California (USA), and they were evaluated at low and high temperatures^28^.

More than 75% of the phages persisted even after incubation on the ice at around 0 °C. Phages are also vulnerable to higher temperatures. 68-70% of phage particles disintegrate when boiled at 105 °C. Mocé-Llivina et al.^29^ discovered that a thermal treatment inactivates phages in dewatered sludge and raw sewage. The thermal resistance of somatic coliphages, which are phages capable of infecting *Bacteriodes fragilis*, and F-specific RNA phages was discovered. This study suggests that phages are more resistant to thermal treatment than bacteria. The most significant parameter in regards to determining phage activity is the storage temperature. *Bacillus cereus* CP-51 phages were sensitive to low temperatures and stable at room temperature, even though phage storage at room temperature is impossible. Tailed phages are the most resistant to storage and have the most extended longevity. Some phages, such as T4, T5, and T7 were viable after 10–20 years at 4 °C. Phages generally resist freezing and thawing, so repeated short-term treatments can antagonistically affect them. Olson et al.^26^ discovered that 4 °C (*k* 40days) in wastewater is the best temperature for phage storage. The temperature must be kept below −80 °C in order to retain the phage activity for a longer period^26^. The phage viability is nil after 84 days in an SM buffer at 42 °C, whereas no phage activity was found after 120 days at 37 °C. According to Hatch and Warren,^30^ phages should not be stored below −20 °C, because ice crystals form at this temperature, which can kill phages.

### pH of the environment

Another critical factor that regulates phage activity is the environment’s acidity. Scientists investigated the presence of phages in wine, particularly those associated with the lactic acid bacterium *Leuconostocoenos*. According to Lu et al.^31^ phages can grow in an acidic environment, such as in sauerkraut. After 60 and 100 days in a sauerkraut fermentation tank, 24 phages were identified (pH 3.5). Kerby et al.^32^ investigated T7 phage stability in several pH (3–11) buffers, which included citrate, phosphate, phosphate–borate, borate buffers, and citrate– phosphate, for 1–2 weeks at 0.5–2 °C. The optimal pH for phage physical stability for long storage is between 6 and 8. The T7 phage is most active at pH 7, and it has the best stability in a phosphate buffer, which only loses 20% of its activity. It was unstable at pH 4 and lost most of its infectivity after 96 h in citrate or citrate–phosphate buffers. Also, it entirely lost its activity after 1 h at pH 3. T7 phages demonstrate at least 30% activity at pH 9 in alkaline conditions, and their infectivity lasts for 15 days. The T7 activity was almost eliminated after 24 h in a borate buffer with a pH >10. Their activity was limited by a pH <3.5 and a total concentration of SO_2_ of 50 mg/L. The phage is generally stable in the pH range of 5–9, with an optimal pH of 5–6. Their instant coagulation occurred at pH 2, whereas the phages precipitated at pH 3 and 4. However, it was alterable at the greater value, and the phages could be redisposed by shaking them. The researchers found that irreversible coagulation and precipitation might be the limiting factor of the phage activity. A little loss of infectivity nearby at pH 7 was also observed. The PM2 phage was sensitive at a low pH, completely losing activity at pH 5.0. Particles of the T1 phage vanished at pH 3.0, and the M13 phage survived even at pH 2. These interpretations show that the alteration in environmental pH may shelter the phage activity at a low temperature.

Wick et al.^33^ reported that MS2 could survive in 0.1 mol/L HNO_3_ for 66 h without losing phage particles. Höglund et al.^34^ studied bacterial viruses in source-separated urine, concluding that these viruses could be found there (pH 9). Inactivation was roughly twice as high at 20 °C as at 5 °C in PBS (pH 7.4), which was utilized as a control. The decrease in the phage titer at 20 °C could have been caused by the conversion of urea to ammonia, a component that inactivates viruses. These findings were verified by Vinner et al.^35^. They discovered that urine dilution and a lower incubation temperature increased the stability of phage 28B, MS2, and phiX174. Their findings on T4 phage stability in urine after 4 weeks of incubation at 6 °C and at room temperature in a urine sample indicated no significant fluctuations in the phage titer, which indicates strong viral stability^36^. The MS2 survival is better in diluted or fresh urine than in preserved urine, according to Chandra et al.^37^. The temperature and pH had a more significant impact on the phage inactivation at 30 °C than at 15 °C. The concentration of hydrogen ions alters the phage aggregation when the pH is less than or equal to the phage isoelectric point (pI=3.9). For example, the MS2 phages showed significant potential to aggregate^38^.

### Salinity and ions

Phages have been reported to be inactivated by osmotic stress. According to Whitman and Marshall,^39^ psychrophilic *Pseudomonas* phages showed less perseverance in highly concentrated NaCl and sugar solutions. The vitality of the phage ps1 was reduced by 99% when diluted in 4 mol/L NaCl. However, the viability of the phage wy was only reduced by 26%. on the other hand, a 2-mol/L sucrose solution reduced ps1 by 50% and wy survival 48%. The effectiveness of both phages was reduced by up to 30% in 0.1% citrate on a soft agar medium. Several phages were recovered from different salinities of seawater. Wichels et al.^40^ grouped the phages into three families. 11 belong to the *Myoviridae*, 7 to the *Siphoviridae*, and 4 to the Podoviridae. This site found no DNA structural similarity across phages from different families. In addition, Hidaka.^41^ investigated the stability of five marine phages in various inorganic salt media. All the phages were shown to be more inactivated in a medium enriched with 0.5% NaCl than in the other media. The phages may have been the most active at salt concentrations that were similar to concentrations that are seen in saltwater. Seaman and Day.^42^ extracted phages from a salt plains soil sample. The salinity of the soil varies between 0.3 and 27%, and the salt content of surface water ranges between 4 and 37% across this region.

## Positive aspects of bacteriophages

### Antimicrobial agents

The use of antimicrobials reflected a better understanding of microbial relations in foods at the time of the food preservation strategy formation. Some microflora is beneficial in food from a competitive aspect, whereas native microflora must be eradicated or killed under other circumstances. As a result, broad-spectrum antimicrobials are frequently replaced with more targeted preservatives. This approach is less effective than bio-preservation with bacteriocinogenic lactic acid bacteria. Using phages is another way to change the bacterial ecology actively. d’Herelle found that lytic phages were effective against the dysentery bacillus (*Shigella*) in the faeces of convalescing patients in 1917, which makes him the first to consider phages as a bio-therapeutic option^1^. Phage prophylaxis has been described for treating various human and animal diseases as well as a patented medicinal treatment. Phage therapy could be a better antibiotic option^43^. Phage therapies are also being used to treat *Klebsiella pneumoniae* infection, and the results in the animal studies that comprise of mice models are positive^44^.

### Alternatives to antibiotics

**Fig. 2:**
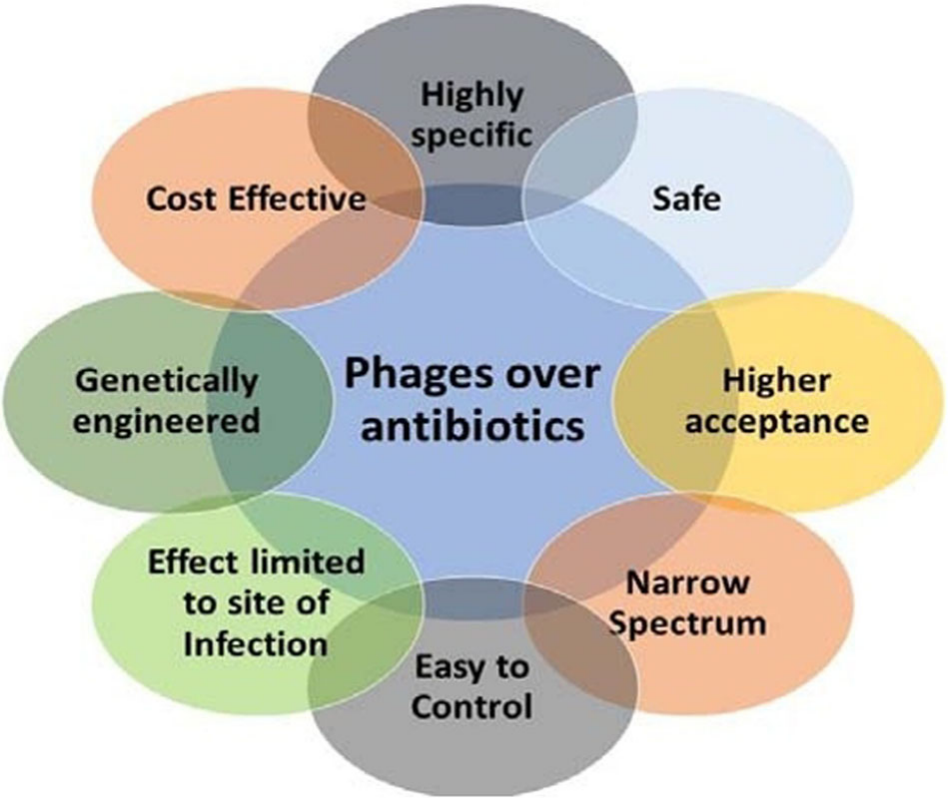
Advantages of bacteriophages over the antibiotics.

Nobody will disagree that the use of antibiotics in various agricultural-based food production should be strictly regulated, because it is a significant source of bacterial resistance that eventually extends to clinical environments. Bacteriophages and phage-isolated proteins (phage therapy) are promising treatments for various bacterial infections^24,45^. Popular antibiotics, such as beta-lactams, tetracyclines, chloramphenicol, and aminoglycosides, are less effective or ineffective in antibiotic-resistant bacteria, causing problems in the medical industry^46^. Almost 700,000 people worldwide die each year as a result of disease-causing bacterium resistance, and fatalities are anticipated to rise by more than 10 million by 2050^47^. The quest for novel medicines is vital, and the battle against antimicrobial resistance is still ongoing. According to experts exploring alternative antibiotic therapy, the most significant challenge is ARGs that code for bacterial resistance to conventional antibiotics, which phages might be a viable option^48,49^. Phages can be used to treat illnesses that are caused by a variety of bacteria, which include *Staphylococcus aureus*, *Pseudomonas aeruginosa*, *Shigella*, and *Salmonella*. It has been shown that many of these bacteria are antibiotic-resistant and can cause fatal Infections^50^. Phages can treat infections that are induced by the lysis phenomena generated by the microorganisms. The west had traditionally lagged in regard to phage therapy development even after successful phage therapy implementation in former Soviet republics and Eastern European nations^13^. Treatment with phages was effective against cystic fibrosis, also known as resistant infections. Some groups of researchers found that bacteriophage treatment is also ineffective on the infectious pathogen, which is due to the resistance against it. Susceptibility assessment of bacteria to a specific bacteriophage is essential before being used as a therapy^51^. Treatment usually consists of a mixture of many phages, which is due to a lack of quick diagnostic screening tests. The bacterial lysis causes the release of endotoxin, which will cause sepsis^52^. Furthermore, the pharmacokinetics of phages easily gets diffused to all other organs. Another key challenge with phage therapy is immunogenicity, which means that the defense system may be triggered the first time and then destroy the phages as soon as they enter the system using the system’s defense mechanism a second time. Phages can now be used in novel ways to prevent bacterial infection, which is due to advances in genetic engineering^53,54^. Antibiotic-resistant infectious diseases of the lungs, which are known as pulmonary infections, are among the most dominant^55^. Phages that can combat these diseases have already been tried in in-vitro models and in animal models, and the results have been promising especially when the phages are delivered via nebulization as in aerosol form^56^. Antibacterial medications, such as phages and antibiotics can be given locally to the lung cells as aerosols for respiratory tract infections^57^. This enables larger concentrations at the site of infection, which prevents antibacterial agent dispersion in places where it isn’t needed. This results in dramatically increased action in situ and fewer potential side effects. A 5-year-old child with cystic fibrosis was given a commercial bacteriophage product after failing to respond to regular antibiotic treatment (prophage) in 2008. The bacteria *P. aeruginosa* and *S. aureus* have been associated with the illness. The drug was delivered three times a day via nasal phage nebulization. No weight gain was observed for a year before the treatment, but the child’s general condition significantly improved after six days of therapy, and a weight gain of 1 kg was observed after twenty days. *S. aureus* and *P. aeruginosa* were undetectable in the sputum after three therapy sessions, which included one with tetracycline^58^. *Pseudomonas* phages demonstrated antibacterial activity in the sputum of cystic fibrosis phages in-vitro^59^. Morello et al.^60^ obtained multi-resistant *P. aeruginosa* from a cystic fibrosis patient and intranasally administered the pathogens to animals in order to induce pneumonia in a mouse model. Bacteria, inflammatory markers, and cytotoxicity, which included cell death and endocytosis, levels were all examined to see how far the infection had progressed. Phage P3-CHA was used in an animal study in order to check the lethal levels of *P. aeruginosa* with two doses of phage 3.0×10^7^ and 3.0×10^8^ plaque forming unit (PFU) per mouse. The bacterial count in the high phage dose treated group was observed to have decreased by more than 2-fold in contrast to the control group 20 h after the treatment. The phage-treated group had significantly lower levels of cytokines and lactic dehydrogenase than the control group. Similar results were seen in the histological investigations of the animals’ lungs. The pneumonia was treated with an intranasal spray of the same phage before infection^61^. Overview of advantages of bacteriophages over the antibiotics are shown in Fig. 2. Aerosol-based phage therapy appears to be a successful way of treating extremely antibiotic-resistant bacterial respiratory infections, particularly infections that are caused by BCC, according to Semler et al.^62^. Another study was conducted in 2015 in order to investigate phage therapy in-vitro and in-vivo in minks against *P. aeruginosa* hemorrhagic pneumonia, as well as the effectiveness of ultrasonic atomization of phage preparations. The researcher used vB PaeP PPAABTNL (PPA-ABTNL), which is a lytic *Podoviridae* phage that is isolated from hospital sewage. The phage was tested against five strains of *P. aeruginosa* that were taken from minks with hemorrhagic pneumonia. The in-vitro tests demonstrated that the phage was exceptionally effective in killing the bacteria it was meant to kill. The phage was later determined to be extremely safe in in-vivo rat investigations. The study states that this delivery method could be used to treat pneumonia caused by these bacteria^63^.

### Control of pathogenic and spoilage bacteria in foods

Food is one of the primary channels of disease transmission, and it contains about 200 well-known diseases. The majority of them are bacterial in origin, which are transmitted through food^64^. Bacteria are responsible for two-thirds of all foodborne illnesses^65^. *Salmonella*, *Campylobacter*, *Escherichia coli*, and *Listeria monocytogenes* are common foodborne pathogens that are usually linked to severe gastrointestinal tract (GIT) infections^66^. These are part of the natural microflora of fresh and unprocessed foods, and these play an important role in maintaining microbial equilibrium in each bacterial ecosystem. *L. monocytogenes* produce listeriosis, which is a fatal disease that is also one of the most common foodborne infections. This opportunistic pathogen is usually associated with fresh and ready-to-eat food contaminated by an infected person, equipment, or factory environment. *L. monocytogenes*, *Campylobacter sp*., and *Salmonella sp*. are rarely seen in animals, even though researchers have concentrated on reducing bacteria in poultry and fresh/processed meals utilizing phages^67,68^.

**Fig. 3:**
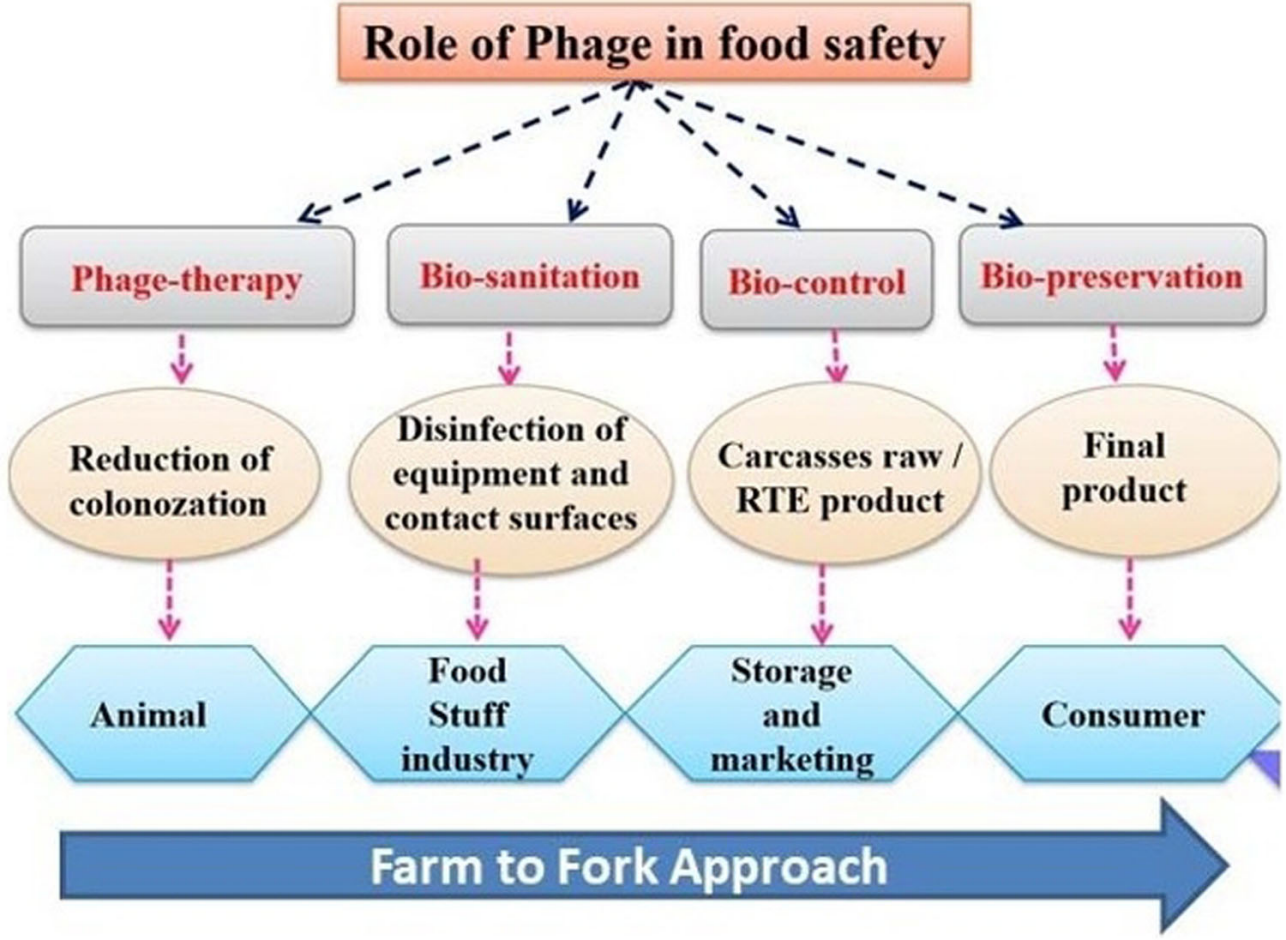
Uses of the phage treatment in the food production chain.

Phages are used in order to improve food quality and yield in the food manufacturing industry, especially in the case of animal-based foods by lowering spreading levels in the atmosphere^69^. Phages are utilized in the food processing industry in order to improve food quality and yield by lowering spreading levels in the atmosphere, particularly in the case of animal-based meals^14^. Temperate phages are frequently viewed as being unfavorable for the development of bio-control strategies. On the other hand, their virulent counterparts (lytic viruses) are suited for bio-control applications. Food-borne pathogen bio-control could be a financially viable use for phage-based bio- control. Several phage-based products have already been approved for use on food products, which include ListShield or LISTEX for the control of *L. monocytogenes*, EcoShield for the management of *E. coli* O157:H7, and SALMONELEX for the control of *Salmonella*^70^. Phages have also been found to be successful at decontaminating livestock that is raised for human consumption by lowering the risk of diseases entering the food supply, which is illustrated in Fig. 3. Infections in animal-derived food products have also been detected using phages.

Research into phage biology and biotechnology, which includes the production of beneficial phage gene products, such as *endolysins* could improve food safety and agricultural output^21^. Several other detailed evaluations summarize the current and potential phage applications across the food chain^71^. Furthermore, the growth in antibiotic-resistant bacteria has sparked and motivated a search for new food bio-preservation techniques^72^. Pre-harvest and post-harvest phage investigations have focused on many life-threatening infections, such as *E. coli* O157:H7, which was documented by Sillankorva et al.^73^.

*Shigella* is a bacterium that can be transmitted via drinking polluted water or eating contaminated food, and it is one of the most common food-borne and water-borne diseases worldwide. Jun et al.^74^ released a study that looked at the possibilities of using a virulent Myoviridae phage (pSs-1) that was isolated from South Korean ambient water as a bio-control agent for *Shigella flexneri* and *Shigella sonnei*-infected waterways. Omnilytics developed the Agriphage to treat bacterial spots that were caused by *Xanthomonas campestris* or bacterial speckles that were caused by *Pseudomonas syringae*^75^. Bacteria are gram-positive, rod-shaped, facultatively anaerobic, catalase-positive, and oxidase-negative. They can be found in a broad range of natural environments, which include water, soil, silage, sewage, animals, and plants. Temperatures that range from 1 to 45 °C with up to 10% salt concentration and pH levels that range from 3.0 to 9.0 are suitable. Individuals become infected with *L. monocytogenes* is an opportunistic pathogen that causes listeriosis^76^. Fever, vomiting, diarrhea, flu-like illnesses, and abdominal pain indicate food contaminated with harmful microorganisms. *L. monocytogenes* is a severe hazard for the food industry due to its propensity to survive and grow in harsh food preservation conditions, such as high salt, acidity, and freezing temperatures. It can regularly be found in almost all raw food sources^77^. A more pronounced reduction in the quantity of *L. monocytogenes* on melon slices was obtained by using a mixture of six phages that were applied at the time of melon processing for 1 h at a concentration of 8 log PFU/ml^78^. Attempts were made to employ phages in foods, such as raw meat, smoked fish, fermented fish, milk, cheeses, fresh-cut fruits and vegetables, and a range of ready-to-eat goods in order to manage *L. monocytogenes*^79,80^.

The researchers could minimize or even remove *L. monocytogenes* from food products in most cases. Most of the trials were conducted with the P100 phage, which was often in the form of the commercial items PhageGuard ListexTM, and several research studies were conducted with the phage mixture ListShield^TM^. Despite the discovery of specialized phages for a variety of bacterial species, which included *Erwiniae*, Phytopathogenic *Pseudomonads*, and *Xanthomonads*, a little amount of research into their effects on plant disease progression, fruit output, and fruit preservation has been conducted^81–83^. Only a few research studies have been done using phages in order to prevent fire blight on apple trees, bacterial spots on tomatoes, and peach bacterial spot disease^83^. Phage bio-control methods are used to inhibit the formation of pathogens that can be hazardous to human health for plant and animal origin foods. There is a lack of data on phage prophylaxis regarding controlling pathogens in meat. *Salmonella* phages have been found to replicate and thrive in the ceca of hens, which helps in order to reduce *Salmonella* species in the stomach and lower mortality^84^. *Campylobacter jejuni* is a highly infectious bacterial infection which is found in large quantities in raw or undercooked poultry. Humans get diarrhea from cross-contaminated items that are made at the same time as the meat, and post-infectious illnesses, such as arthritis and peripheral nerve paralysis, develop in rare cases^85^. Infections caused by *C. jejuni* are rising in both developed and developing countries. *C. jejuni* has developed resistance to many essential antibiotics at the same time, which makes treatment even more difficult. Chemical or heat treatment can minimize *Campylobacter* infestation on chicken carcasses. However, this may alter the taste or look of chicken meat. Other options include injecting anti *C. jejuni* probiotics into live hens or utilizing phages in order to eliminate the bacteria. *Salmonella* is the most common foodborne pathogen, and it is one of the four leading global causes of diarrheal diseases according to the World Health Organization. Infections with *Salmonella* are spread mostly by infected meat, poultry, eggs, and milk. Direct contact with infected animals, blood, urine, and excreta can harm human health. Antibiotics have become more widely used in order to treat infections in livestock and increase food production by accelerating the spread of antimicrobial-resistant bacteria^72^. Antibiotics that are now available are ineffective in regards to treating MDR *Salmonella* infections, which have become a public health concern. Leverentz and a co- worker artificially inoculated a mixture of four different *Salmonella enteritidis* phages in melon slices during refrigeration storage and found a significant reduction in the *salmonellae* counts. A mixture of four different phages of *Salmonella enteritidis* resulted in substantial decreases in the number of salmonellae recovered during storage and abusive temperatures from artificially inoculated melon slices^86^.

### Potential biodetectors

Phage-based sensors have received a lot of interest due to their high specificity, sensitivity, and simplicity. Phages are emerging as novel actors in the quick and specific detection of microbes^87^. Phages are easily immobilized on electrode surfaces due to the abundance of active components on their surfaces. Phages only infect specific bacterial strains, so phage-originated or phage-mixture recognition proteins can be designed in order to sense the target bacterial spectrum in their natural state selectively^88^. A single phage can target a specific bacterial species or even a strain, so a phage that recognizes the target bacteria can always be found^89^ Whole-phage probes, nucleic acid probes, phage-display peptides, antibodies-based probes, and receptor-binding proteins from phages have all been used in order to detect infections on biosensor surfaces^90^. Bioassays and biosensors based on phages have been developed using surface plasmon resonance, magnetoelastic platform, quartz crystal microbalance, and electrochemical techniques. Surface plasmon resonance and surface-enhanced Raman spectroscopy transducers are widely used in developing phage-based bacterial detection systems^91^. For example, a silicon surface was delicately melded on a thin sheet of silver before being immobilized by the T4 phage after being treated with glutaraldehyde and 4-aminothiophenol. The surfaces were paired with the surface-enhanced Raman scattering approach for increased bacterial sensitivity with a detection limit of 100 log CFU (Colony forming unit)/mL^92^. Phage-based techniques can also be employed for the detection and antibody selection of coronaviruses^93^.

### Phage-derived lytic proteins

Phage endolysins, such as peptidoglycan hydrolases, are emerging as a promising source of biologically active agents to severe infections and other undesirable contaminations. Peptidoglycan hydrolases are endolysins that are released near the end of a phage’s lytic multiplication cycle in order to lyse the host cell via a peptidoglycan breakdown^94^. In this *lysis-from-within* mechanism, holin proteins allow endolysins to permeate the cytoplasmic membrane and access their substrate^95^. Compared to the control, endolysin, such as LysH5 effectively killed *S. aureus* in cow milk, which reduced the quantity of CFUs by 8 log. Combining LysH5 with the bacteriocin nisin could increase this activity further by leveraging a considerable synergistic impact between these two antibacterial drugs with fundamentally different modes of action. Moreover, phage lytic enzymes, such as endolysins and virion-associated peptidoglycan hydrolases are a family of antibiotics known as enzybiotics with a diverse set of characteristics^96^. In addition to their strong lytic capacity, selectivity, and modular structure, bacteria resistant to enzybiotics have not yet been discovered^97^.

### Application in the food industry

A phage is most likely employed as an antibacterial as well as a preservative in food production and processing in order to prevent pathogenic contaminants. Food quality is a major concern in the food industry, because it directly affects human health. Food serves as a vehicle for disease-causing microbes to travel from *farm to fork*^45^. Phages are also known as a powerful bio-control tool in the food business, where they target harmful bacteria. Many studies revealed that phages are safe for humans, animals, plants, and the environment, but some literature still has contraventions^98^.

### Biofilm control mechanism

Pathogenic bacteria proliferate and create a biofilm on equipment due to poor cleaning, which pollutes the product, in the dairy sector. It also serves as a vehicle for spreading an illness. Bacteria are encased in a matrix that shields them from the harsh environment, which makes it difficult to remove or clean off surfaces. A staphylococcal phage was isolated in the lab that efficiently combats staphylococcus biofilm and actively removes it. Biofilm reduction is higher at 37 °C for 72 h in the case of phage K, and a mixture of the derivatives is better at 37 °C for 72 h. Phage K and another staphylococcal combination completely remove the biofilm after 48 h at 37 °C^99^. *E. coli* forms a biofilm on food processing surfaces, such as stainless steel, ceramic tile, and high-density polyethylene, and the number of *E. coli* drops below the detection level after treatment with a phage mixture called BEC8^100^. Phage P100 can minimize the biofilm development on stainless steel by *L. monocytogenes* by up to 5.4 log/cm2^101^. Some industrial manufacturers designed and developed a phage-based solution to prevent and disinfect foodborne infections. ListShield^TM^ and PhageGuard Listex, which are the first commercialized phage-based products, are employed in the food sector. PhageGuard Listex can be sprayed or immersed in order to prevent *Listeria* contamination of cheese, and it has little or no influence on the product’s color, texture, or flavor. *Pseudomonads* of milk origin is resistant to phages that are isolated from raw beef^102^. The researchers discovered that the phage can reduce the amount of *Salmonella* in cheese and chicken in the absence of bacterial growth, which is shown in Table 1^103^.

**Table 1:** Application of bacteriophages and their mixture for the removal of pathogenic and spoilage bacterial biofilms.

Spoilage causing bacteria/ pathogens	Phage(s) mixture	Application	Phage efficiency	References
*B. licheniformis* (10^8^ CFU/ml)	FBL1	Glass	Biofilm recovery 0.79% after 48 h	^139^
*E. coli* EPEC 920 (10^3^ CFU/ml)	DT1 (10^8^ pfu/ml)	Milk fermentation	1.1 log reduction after 24 h at 37 °C	^140^
*E. coli O157:H7* (10^6^ CFU/ml)	BEC8 (Phage mixture)	Stainless steel, ceramic tile, and high-density polyethylene	Biofilm-forming cell counts are untraceable after 1 h of treatment at 12, 23, and 37 °C	^141^
*L. monocytogenes* (10^4^ CFU/cm2)	Phages LiMN4L (10^9^ pfu/ml)	Stainless steel coupon	Bacterial Biofilm invisible after 75 min	^142^
*S. aureus* (10^6^ CFU/ml)	DRA88 and phage K (10^9^ pfu/ml)	Polystyrene	Removal of the biomass after 48 h	^99^
*S. aureus* (10^6^ CFU/ml)	PhiIPLA-C1C and phages phiIPLA-RODI and (10^9^ pfu/ml each)	Polystyrene	Reduction by 2 log units/well after 8 h of treatment	^143^
*S. aureus* (10^6^ CFU/ml)	SANF (10^8^ pfu/ml)	Commercial pasteurized Milk	96% after 6 h at 5 °C	^144^
*S. aureus* Sa9 (10^4^ CFU/ml)	Phage (ΦIPLA35, ΦIPLA88) (10^8^ pfu/ml)	Pasteurized whole milk	Complete decline, 24 h at 25 °C	^68^
*S. aureus* Sa9 (10^2^ CFU/ml)	Phage (ΦH5 and ΦA72) (10^8^ pfu/ml)	UHT milk	Complete decline after 2 h at 37 °C	^100^

### Application in the gastrointestinal tract

The human GIT is thought to contain 10^15^ phages, which makes it the planet’s highest concentration of biological creatures. The three virus families with the highest frequency and abundance in the human GIT are Podoviridae, Siphoviridae, and Myoviridae^104^. The Republic of Georgia’s Eliava Institute of Phage, Microbiology, and Virology published the first reports of phage therapy for intestinal illnesses in the 1930s^105^. The initial reports about the use of phages in the fight against cholera infections were promising. Three classes of the phage community are found in the human gut that can maintain a balance between the gut microbiota, which are responsible for health and disease conditions^106^.

There is limited and inadequate scientific data from this time period, but the evidence suggests that prophylactic phage therapy decreased dysentery outbreaks in Soviet soldiers. Soldiers who received phage therapy had a 10-fold lower prevalence of dysentery outbreaks than soldiers who did not have the phage therapy^107^. According to estimates, around 30,000 children in Georgia were treated with tableted phage therapy for dysentery in the 1960s. The control and treated groups were separated by the street that they lived on. The children who had taken the phage therapy and were treated with it lived on one side of the street, whereas the children who lived on the other side of the street were treated with the placebo treatment. Their findings revealed a 3.8-fold decline in dysentery episodes among the street-side children who were the participants^107^. IntestiPhage formulations are phage mixtures that are used to treat and prevent infections that are caused by *Staphylococcus, E. coli, Shigella, Salmonella, Pseudomonas*, and *Proteus* in the intestines. An interesting report on the use of IntestiPhage in youngsters from 1976- 1982 was released by Kutateladze and Adamia^58^. 452 children were given the IntestiPhage preparation, 100 children were given antibiotics, and 28 children were given both antibiotics and the IntestiPhage preparation. The antibiotic-treated group exhibited clinical improvement after an average of 29 days. The phage-antibiotic combination group exhibited clinical improvement after 15 days, but the IntestiPhage preparation alone recovered in only nine days on average^58^. A *randomized, double-blind, placebo-controlled study* was conducted to determine the efficacy and safety of phage mixtures. A 9-phage mixture, which included nine different isolates of T4-like *E. coli* Phages that comprised of four phages highly connected with the T4D reference phage, was given to 15 healthy people. The participants received either 3.0 × 10^7^ PFU per person or 3.0×10^9^ PFU per person. The doses were given three times a day, and were diluted in 150 mL of mineral water. The placebo group simply received mineral water as a treatment^108^. No treated volunteers showed any clinical adverse effects, according to reports, medical examinations, lab tests for kidney and liver functions, and hematological assays. Another prevalent cause of food poisoning, which includes gastroenteritis, is *C. jejuni*, which infects at least 2 million people yearly^109^. One of the main reasons for *C. jejuni*’s pathogenicity is its tendency to form biofilms, which makes antibiotics less effective and eliminating *C. jejuni* infections more difficult^110^. Siringan et al.^109^ examined the effects of two phages, which included CP8 and CP30, on biofilms that were formed on a glass surface by *C. jejuni* strains NCTC 11168 and PT14. Phages reduced the number of viable bacteria per cm2 from 1 to 3 log10 CFU/cm2. The viruses within the biofilm were able to kill and lyse campylobacters, and they were also able to propagate the extracellular biofilm-producing matrix. Gastric acidity can destroy phages, so the route of administration is especially important in phage therapy for GIT infections. Microencapsulation was highly effective for the oral administration of these types of phages^111^. Combining phages and probiotics provides an alternative treatment for dysbiotic diseases of the human gut. Deresinski^12^ observed this relationship in leukemic patients with dysentery. The researchers used four different therapies on 59 patients with this illness. The first group received oral phage therapy, *Pseudomonas* phage or *Proteus-E. coli* phage. The second group received a probiotic (bifidobacteria). The third experimental group received a mixture of phages and *Bifidobacteria*. Antibiotics were administered to the fourth group as a typical oral medication. Clinical effects were the highest in the group that got a combined therapy of phages and probiotics.

### Application in biofilm destruction

- Phages replicate within their host cells, which increases the localized phage population (amplification). Infectious phages are released and penetrate the biofilm.
- Phages propagate throughout the biofilm and kill exopolysaccharide-producing bacteria, which remove the biofilm and reduce the chance of regeneration.
- Phages may transport or express depolymerizing enzymes that destroy the exopolysaccharide from within the host genome.Phages can infect the persister cell even if they are unable to reproduce and destroy the inactive cell. These remain inside the cell until they become reactive and form a vegetative cell, which begins to multiply and destroy the cell via lytic action afterward. If a large number of phages are present, they can kill their target host cells without replicating^110,112,113^. However, these types of cases are uncommon, and obtaining large numbers like this in the lab is difficult. A smaller number of phages are utilized to replicate, kill the host cell, destroy the host cell, and then repeat the cycle with a larger number of bacteria in the lysis cycle. There are not enough host cells, so this cycle is disrupted and interrupted. Biofilms are quite frequent and contain many bacteria, so the phage’s successful targeting of bacteria within biofilms likely represents an evolutionary change to use this abundant source. Their mechanisms for doing this are thought to be based on their need to deal with bacterial capsular polysaccharides throughout the usual course of an illness.

Many phage genomes contain genes for depolymerizing enzymes that can break down the biofilm matrix. These soluble enzymes that target bacteria by breaking their cell walls are released from the host cell. These enzymes also can affect and degrade the exopolysaccharide in the biofilm. The host cell degradation releases the DNA, which remains attached to the biofilm formation. Phages require the tail within the enzyme for infection, which is a general model of tail phages. Capsular polysaccharides are recognized and digested by a phage tail component in this scenario, which allows the tail to access cell membranes and inject the bacterial genome^114,115^.

## Negative aspects of bacteriophages

The use and effectiveness of phage therapy in humans, animals, and plants have been previously demonstrated, but there are some limitations as well as potential issues when utilizing phage treatment therapy in other circumstances, which include fundamental human safety issues^116^. No harmful effects have been observed due to phage treatment, but the purity of phages may pose a problem. Phages emit lipopolysaccharides, peptidoglycan, or other inflammatory components after they lyse bacteria, which can end up in the crude phage preparation^117^. Various technologies, such as density gradient centrifugation and column chromatography^118^, are now available for phage purification that are simple and cost-effective, and these methods have reduced problems that are related to contaminants. Phage contamination during fermentation gave the first evidence in the dairy industry, which provided crucial information on the presence of phages in the food industry. These dietary settings serve as a host for bacteria and phages to coexist. Many factors may limit the use of phages and the creation of new therapeutic formulations. Furthermore, preparing phages for medicinal application is challenging, and not all of the issues that are strictly interrelated with phage biology have been resolved^6,119,120^.

### Increased risk of antibiotic resistance

Lysogenic phages could be vehicles for horizontal gene transfer and contribute to ARG spread. Transduction might theoretically result in the emergence of new microbes or even more resistance genes in bacteria^121^. However, the exact role of phages in the propagation of ARGs remains unknown. Phage inducers, which are substances that are capable of encouraging the expression of the prophage gene or leading to the excision and spread of temperate phages, can help disperse ARGs in the environment. Bacteria can be infected with new phage particles and then be lysed with EDTA or sodium citrate, which triggers the lytic cycle of lysogenic viruses and phage release outside the cell. Antimicrobial-resistant pathogen-infected patients’ secretions and tissues have a substantial number 600 of phage transport genes that are linked to antibiotic resistance that has previously been constantly treated with 601 antibiotics^49^. Some antibiotic-resistant genes have been reported in the phages present in the environment, but its efficacy to transfer these genes to the bacteria and the resultant successful uptake and expression of these antibiotic-resistant genes needs to be further studied before taking any conclusive inferences^122^.

### Impact of phages on the food industry

The role of phages in starter culture failure has been extensively researched and discussed^70^. More than 10% of the raw milk samples that were collected from different dairies included *Lactococcus lactis* phages. 37% of the lactococcal and streptococcal phages were identified in the raw milk samples that were used for yoghurt manufacturing in another study. Refrigerated foods, such as red meats and poultry, are perishable, and they support a complex microbial environment and huge numbers of bacteria (2 to 6 log CFU/g). These dietary conditions serve as hosts for bacteria and phages to coexist. The most common cause of fermentation failures in the dairy industry is the phage infection of dairy starter cultures. Phage breakouts can result in financial losses, such as factory halts, raw material waste, poor product quality, microorganism proliferation, or even total production loss^15^. The starter activity is completely lost due to a dead vat when a phage infection is severe, which results in the dumping of a considerable amount of partially acidified milk. A few actions should be taken to avoid the adverse effects of phages on dairy starting culture, which include the use of mixed strains, the cheese manufacturing and whey handling unit must be located away from the starter preparation area, the use of a phage inhibitory medium for bulk batch starter production, the injection of frozen concentrated starter cultures directly into cheese vats, sanitation regimes, air filtering, and a variety of additional tactics. The effective monitoring at the area’s entrance, swift and effective phage detection tools, and control measures help limit the risk of phage proliferation within the fermented sector of the dairy industry.

Natural phages may impact the variety of microbial communities by exerting species-specific control over indigenous bacteria, thereby influencing the microbial communities’ diversity^123^. The close monitoring of entrance channels, quick and effective phage detection technologies, and control measurements are being used in order to limit the risk of phage proliferation in dairy settings. Manufacturing delays, the waste of components, lower quality products, the growth of spoilage, and infectious microbes, or even total production loss, can all result from phage outbreaks^15^.

### Bacterial resistance against phages

Bacteria potentially and possibly may become resistant to phages over a specific period, and bacteria have or can create a multitude of methods in order to evade phage attacks. These mechanisms primarily prohibit phage adherence to bacterial receptors, the activation of steps to prevent phage DNA inoculation into the cell, and the prevention of phage reproduction and release by hiding, altering, or losing the receptor and secretion of chemicals^124^. Variation or the lack of receptors for membrane protein changes is demonstrated in *E. coli*, *S. aureus*, *Bordetella bronchiseptica*, and *Vibrio cholerae*. *Pseudomonas spp* release extracellular polymeric compounds, and Enterobacteriaceae release glycoconjugates^125,126^. The antibiotic association, a mixture of phages that are capable of lowering the bacterial resistance, or a high concentration of original phages are all viable options. The phage mixture, a higher initial phage inoculum, and antibiotic interactions can all help in order to reduce the development of *bacterial resistance* to phages. If phages eliminate pathogens quicker than these can multiply, a large inoculum is linked to a lower likelihood of generating phage-resistant bacteria^11^.

## Legal framework for bacteriophages

The search for alternatives to antibiotics is critical since antimicrobial resistance has grown to be a worldwide concern. Bacteriophage therapy has emerged as a viable substitute for treating bacterial illnesses resistant to many drugs. The European Medicines Agency (EMA) considers natural bacteriophages used as therapies to be medical products^127^, classifying them as biologicals under Directive 2001/83/EC on the Community law related to medicinal products for human use^128^. Although many nations have only recently regained their interest in phage treatment due to the AMR epidemic, it has long been employed in Eastern Europe. Phage treatment, in particular, has been used in health care in Georgia, Poland, and Russia since its discovery^129^. According to several Polish laws, including the Medical and Dental Professions Act of December 5, 1996, the Polish Association’s ethical code, the Polish Constitution, and EU laws pertaining to its member states, phage therapy was regarded as an experimental treatment in Poland^130^. In Poland, phage treatment is also governed by Directives 2001/20/EC and 2005/28/EC, which govern clinical studies and Good Clinical Practice^131^. Phage therapy has been used for a long time in Eastern Europe, but its usage is more dispersed in Western European countries, including the UK, France, and Belgium. However, recent developments in these nations have shown significant improvements in the legislation governing the treatment. Since 2011, the European Medicines Agency has classified phage therapy as a medicinal product. However, disagreements have arisen regarding whether the classification should be biological medicinal products under Commission Directive 2001/83/EC or advanced therapy medicinal products under Commission Directive 2003/63/EC^132^. Although phage treatment is not licensed in the United Kingdom, many parts are regulated by the Medicine and Healthcare Products Regulatory Agency. This agency also oversees the compassionate use of phage therapy and classifies natural phage as biological medicine^133^. Phage treatment has also gained popularity in Australia in recent years among researchers and medical professionals. Researchers and clinicians in hospitals and research institutes nationwide are connected by Phage Australia, a national collaboration aiming to systematize phage therapy. It is not yet easily accessible to the general public, yet stakeholders strive to professionalize the therapy^134^. Phage treatment was categorized as a biological product by the FDA’s Office of Vaccines Research and Review in the Center for Biologics Evaluation and Research, and as such, it is subject to regulations and production that include GMP, preclinical research, and clinical trial documentation^135^. Even though no FDA-approved phage treatment is currently available, the United States has the most phage-related industry-sponsored trials (ISTs), some of which are in the phase 3 clinical trial stage. With the Indian Council of Medical Research (ICMR) bringing together phage researchers and stakeholders to discuss pertinent details, the Indian government is attempting to promote phage therapy after realizing its potential. As a result, more specialized regulations and research centers can be anticipated in India^136^. The Electronic Code of Federal Regulations (eCFR-FDA) has listed *Listeria*-specific bacteriophage preparation for *Listeria monocytogenes* as an antimicrobial agent (additive) in accordance with current good manufacturing practice. To control *L. monocytogenes* by direct application to meat and poultry products that comply with the ready-to-eat (RTE) definition in 9 CFR 430.1. However, current good manufacturing practice is consistent with direct spray application of the additive at a rate of approximately 1 mL of the additive per 500 cm2 product surface area^137^.

## Phage mixtures and commercially available products

The phage mixture composition is crucial to phage therapy’s success. Phage therapy can only be successful if the right 644 composition and number of phages are used. Designing antibiotic combination therapy is simple compared to 645 constructing a phage mixture. Changing phage mixtures for each infection is time-consuming and expensive, and 646 broad-spectrum phage therapy will not deliver a specific outcome for a particular strain, which is due to adverse 647 clinical effects^13^. The effect of phages on the native microbiota is a topic that has not yet been thoroughly investigated. The gut virome, which compares lysogenic phages to the native gut microbiota, appears to be extremely frequent. Lysogenic phages can effectively suppress virulence genes in pathogenic bacteria. It contains genes for short-chain metabolisms, such as fatty acids, virulence genes, and ARGs. ARGs were detected in phages from human faeces, and a substantial number of phage-encoded ARGs were found in mice treated with antibiotics, indicating that phages could serve as a reservoir for ARGs. Several industries have produced phage-based products for use in food safety applications. Intralytix Inc. created three 654 phage-based products, which include ListShield^TM^, EcoShield^TM^, and SalmoFresh^TM^. The FDA approved these 655 products for use as food additives. In addition, two phages for veterinary applications were also planned, which include 656 PLSV-1^TM^ and INT-401^TM^. This company’s phage products are approved for use in poultry for animal health, of which 657 are actively active against *salmonella* (PLSV-1^TM^) and *clostridium perfringens* (INT-401^TM’^)^138^.

From the first classification as a human enemy, it has already been revealed that phages play a key role in biotechnology, the environment, industry, and medicine. These are often isolated from various sources, and various environmental conditions, including temperature, pH of the environment, salinity of the medium, and ionic concentration, influence their development. Phage treatment can potentially serve as a substitute antimicrobial agent for antibiotic therapy. Phage-based biocontrol chemicals can also be employed to regulate different food-spoiling and harmful microorganisms selectively. Phage lytic enzymes, specifically endolysins and peptidoglycan hydrolases, have gained attention as potential therapeutic agents, and they are commonly referred to as enzybiotics. These enzymes are derived from bacteriophages (phages), viruses infecting bacteria. Research in this field is ongoing, and the development of enzybiotics as therapeutic agents is an exciting area of study in the quest for new and effective treatments against bacterial infections, especially those caused by antibiotic-resistant strains. Bacteriophages, or phages for short, have been explored and utilized as efficient biodetectors to monitor and detect undesired microbial pathogens in various settings, including foods and medicines. Overall, phage-based biodetection represents a valuable approach for monitoring and ensuring the safety of multiple products, including foods and medicines. It’s important to note that the design of phage mixtures for therapeutic use requires careful consideration, including understanding the target bacteria’s specific characteristics, the infection’s dynamics, and the potential interactions between different phages. Research in phage therapy continues to explore optimal strategies for designing effective phage mixtures to combat bacterial infections. The commercialization of phage-based products has been a notable development in the field of microbiology and biotechnology. Several phage-based products have been explored, developed, and, in some cases, successfully marketed as therapies or medicines. These products leverage the unique properties of bacteriophages to treat bacterial infections. In conclusion, addressing the challenge of phage resistance is pivotal for the long-term success and sustainability of phage-based applications. Ongoing research, technological innovation, and a holistic understanding of the dynamics between phages and bacteria are essential for developing effective strategies to overcome and minimize resistance in future applications.

### Reporting summary

Further information on research design is available in the Nature Research Reporting Summary linked to this article.

### Supplementary information

Reporting Summary

## Data Availability

Data sharing is not applicable. This is a review article and no new datasets were generated or analyzed during this study.
